# Testing immune priming failure in laryngeal squamous cell carcinoma: direct evidence, unresolved links, and a compartment-resolved framework

**DOI:** 10.3389/fimmu.2026.1982997

**Published:** 2026-09-28

**Authors:** Wei-Zhe Hong, Guan-Jiang Huang, Qi-Ping Luo, Biao-Qing Lu

**Affiliations:** 1 Department of Otorhinolaryngology Head and Neck Surgery, Zhongshan Hospital of Traditional Chinese Medicine, Affiliated to Guangzhou University of Traditional Chinese Medicine, Zhongshan, Guangdong, China; 2 The Tenth Clinical Medical College of Guangzhou University of Traditional Chinese Medicine, Zhongshan, Guangdong, China

**Keywords:** antigen presentation, cGAS-STING, cross-presentation, dendritic cells, HLA-DR, immune checkpoint resistance, laryngeal squamous cell carcinoma, tumor-draining lymph nodes

## Abstract

Direct evidence for a continuous immune-priming defect in laryngeal squamous cell carcinoma (LSCC) remains incomplete. This Review therefore does not treat failed priming as an established LSCC mechanism. Instead, it separates direct LSCC observations from evidence derived from mixed-site head and neck squamous cell carcinoma cohorts and mechanistic studies in other tumors, using this hierarchy to identify supported pathway nodes and unresolved connections. Direct LSCC studies support recurrent loss of MHC class I antigen-processing components, favorable associations between tumor HLA-DR and clinical outcome, accumulation of PD-L2-positive plasmacytoid dendritic cells, remodeling of dendritic cell states, and an association between tumor-cell STING abundance and advanced disease. None of these findings demonstrates antigen-specific priming. Cross-tumor evidence provides mechanistic context for cDC1 cross-presentation, CCR7-dependent migration, tumor-draining lymph-node priming, and functional cGAS-STING signaling, but these links require LSCC-specific validation. We therefore distinguish MHC class I from MHC class II presentation, cross-presentation from tumor-cell HLA-DR expression, lymph-node priming from intratumoral restimulation, and STING abundance from activation, downstream output, and function. Translational priorities are grouped into evidence-based tiers according to the strength of LSCC evidence and intervention tractability. The MHC-I antigen-processing machinery, PD-L2-positive pDC states, and functional cGAS-STING competence merit the earliest LSCC-specific validation, whereas CLEC9A-, XCR1-, and FLT3L-directed cDC1 strategies remain mechanistically compelling but rely mainly on evidence from other tumor systems. Future multicenter studies should incorporate HLA genotype, population diversity, paired tumor and draining-node sampling, functional assays, and bioinformatic or artificial-intelligence integration with external validation. The proposed sensing-low, presentation-deficient, regulatory-DC-dominant, and signal-present but functionally disconnected phenotypes remain research constructs rather than clinical selection tools. The framework is intended to test where priming fails, if it fails at all, without presenting an unproven LSCC-specific pathway as established biology.

## 1 Introduction

Laryngeal squamous cell carcinoma (LSCC) presents an unusual immunological problem. It arises in a mucosal organ that is repeatedly exposed to inhaled particles, microbial products, tobacco-associated injury, and treatment-related tissue damage, yet clinically meaningful antitumor immunity is not assured. This Review treats checkpoint inhibitor evidence from recurrent or metastatic head and neck squamous cell carcinoma (HNSCC) as contextual evidence from an anatomically heterogeneous population rather than as an LSCC-specific estimate of efficacy. This distinction matters because HPV-negative HNSCCs from different anatomical sites have non-equivalent genomic, transcriptional, hypoxic, and immune profiles (1). More recent profiling of recurrent or metastatic HNSCC also indicates that anatomical site and the number of prior treatment lines can shape the molecular and immune landscape (2). Direct LSCC studies point to defects at earlier stages of the cancer immunity cycle. Downregulation of HLA class I and several antigen-processing machinery components has been associated with reduced CD8-positive T cell infiltration and unfavorable survival (3). A separate immunohistochemical series also reported frequent loss or reduction of TAP1, LMP7, and HLA class I (4). These findings justify closer examination of antigen display in LSCC. They do not, however, indicate a uniform presentation defect across tumors or a single cause of treatment resistance.

The usual description of an inflamed or non-inflamed tumor based on tumor-infiltrating lymphocyte density cannot resolve this problem. T cell presence does not necessarily indicate tumor antigen recognition, and antigen recognition does not necessarily reflect productive priming or subsequent effector killing. A tumor section containing CD8-positive cells cannot identify the peptides recognized by their T cell receptors or determine where these cells first encountered antigen. Nor can it show that a professional antigen-presenting cell supplied adequate costimulation and differentiation signals. LSCC observations illustrate this interpretive gap. Tumor cell HLA-DR expression has been associated with better clinical outcomes in two cohorts (5, 6). In one 56-patient series, HLA-DR was detected on tumor cells in 18 cases and was associated with favorable disease-specific and relapse-free survival, although the imprecision of some hazard estimates limits strong inference (5). A later study linked high tumor HLA-DR expression to greater CD4-positive T cell infiltration and favorable outcomes (6). These associations may reflect an interferon-exposed or otherwise inflamed tumor state, or a functionally relevant MHC class II program. Neither possibility has been confirmed by evidence of peptide loading, antigen-specific CD4-positive T cell activation, or *de novo* priming of naive T cells. Mixed-site HNSCC data strengthen the plausibility of the biomarker hypothesis because a malignant cell interferon and MHC class II program was associated with pembrolizumab response (7). Such evidence is generalizable in principle but not specific to LSCC and cannot by itself validate tumor HLA-DR as a predictive biomarker in laryngeal cancer.

Observations involving dendritic cells and innate sensing provide further evidence of disconnected immune states. In LSCC, increased tumor-infiltrating plasmacytoid dendritic cells were associated with nodal disease, adverse outcome, and lower CD8-positive T cell infiltration (8). The same study found a higher proportion of PD-L2-positive pDCs in tumor than in paired adjacent non-tumor tissue and an inverse association between PD-L2-positive pDC abundance and CD8-positive T cell infiltration, but it did not test causality (8). Single-cell studies of vocal cord leukoplakia and LSCC have identified immune remodeling before or during malignant transition (9). One analysis reported abundant LAMP3-positive dendritic cells in leukoplakia and correlations with regulatory T cells and exhausted CD8-positive T cell states (10). LAMP3 expression alone does not prove lineage origin, migration to tumor-draining lymph nodes, or suppressive function. Likewise, tumor cell STING abundance was associated with advanced stage, higher grade, and nodal positivity in a 59-sample LSCC series (11). Protein abundance is not equivalent to recent pathway activation, downstream interferon output, or effective dendritic cell cross-priming. A detectable immune pathway may nevertheless remain disconnected from antigen-specific T cell immunity. Spatial evidence from mixed-site HNSCC offers a useful comparison. CCR7-positive dendritic cell niches were associated with survival in a pretreatment pembrolizumab cohort (12). However, comparable evidence for LSCC-specific dendritic cell trafficking and lymph node priming has not been identified in the current literature.

This Review addresses a question that lies upstream of the dominant explanations for immune failure in LSCC. Two recent companion reviews examined cancer-associated fibroblasts and myeloid-derived suppressor cells as mediators of stromal remodeling, immune exclusion, myeloid suppression, and checkpoint inhibitor resistance (13, 14). The present Review does not duplicate those downstream frameworks, nor does it assume that LSCC possesses a unique or uniform failure-to-prime phenotype. Instead, it asks whether danger sensing, antigen processing, peptide presentation, dendritic cell maturation, lymphatic migration, and tumor-draining lymph-node priming are functionally connected in LSCC. Tumor-intrinsic MHC class I presentation is separated from MHC class II presentation, cross-presentation from tumor-cell HLA-DR expression, naive T cell priming from intratumoral restimulation, and STING expression from activation, output, and function. Direct LSCC observations are treated as the primary evidence layer. Mixed-site HNSCC studies are used as anatomical and clinical context, whereas studies in other tumors and experimental systems are used only to establish mechanistic plausibility. This hierarchy is intended to prevent cross-tumor mechanisms from being presented as LSCC findings. The central model is deliberately conditional: in some LSCCs, detectable innate signals and HLA molecules may remain disconnected from competent antigen processing, professional dendritic-cell activation, migration to draining lymph nodes, and tumor-specific T cell priming. “Failure to prime” is therefore a falsifiable organizing framework rather than an established disease mechanism. The purpose of the Review is to define which nodes are supported in LSCC, which links remain untested, and which translational candidates justify prioritized validation.

## 2 A compartment-resolved framework for sensing, presentation, and priming

### 2.1 Step 1: danger sensing is distinct from antigen sensing

The first step is detection of danger, not recognition of a tumor peptide. cGAS detects aberrantly localized double-stranded DNA and generates cyclic GMP-AMP, which engages STING to activate IRF3 and induce type I interferon and NF-κB-associated outputs (15, 16). These signals may promote chemokine production, dendritic cell recruitment, and acquisition of an activated state. They do not identify which tumor antigen will be processed or which T cell receptor will recognize it. This separation is especially important in LSCC because the available direct study measured tumor cell cGAS and STING abundance rather than pathway activation or antigen-specific immunity (11). Innate sensing should therefore be treated as a potentially enabling condition. Its immune consequence depends on the responding cell type, signal duration, downstream competence, and connection to professional antigen-presenting cells.

### 2.2 Step 2: antigen processing and MHC presentation

Antigen visibility requires more than HLA expression. For direct recognition by CD8-positive T cells, tumor-derived proteins must be processed, loaded onto MHC class I molecules, and displayed as peptide-bound MHC complexes at the tumor cell surface. LSCC studies showing loss of HLA class I or antigen-processing machinery components identify plausible defects in this route (3, 4). Cross-presentation is a separate process in which dendritic cells acquire extracellular tumor material and route selected antigens into MHC class I presentation. Across experimental cancer systems, cDC1s are specialized for this function and can cross-prime tumor-specific CD8-positive T cells when antigen display is accompanied by costimulation and appropriate cytokines (17, 18). MHC class II presentation must also be considered separately. HLA-DR-positive LSCC cells may participate in recognition or restimulation of CD4-positive T cells (5, 6), but expression alone does not establish a competent peptide repertoire or the costimulatory capacity required to prime naive CD4-positive T cells. For each route, peptide-bound MHC display, cognate T cell receptor engagement, and the costimulatory context must be evaluated as distinct requirements.

### 2.3 Step 3: dendritic cell maturation, migration, and priming in tumor-draining lymph nodes

Antigen acquisition inside the tumor does not guarantee that dendritic cells complete a productive migratory program. Activated conventional dendritic cell states can arise from more than one lineage and may coexpress stimulatory and inhibitory molecules (19, 20). In mechanistic tumor models, CCR7 enables antigen-bearing dendritic cells to reach tumor-draining lymph nodes, where they stimulate naive T cells directly or transfer antigen to lymph node-resident antigen-presenting cells (21). Recent studies further support tumor-draining lymph nodes as sites where migratory cDC1s sustain therapeutically relevant stem-like CD8-positive T cell populations (22). These observations establish biological plausibility, not an LSCC-specific pathway. The current LSCC literature set does not directly trace antigen-bearing dendritic cells from the primary tumor to a draining cervical lymph node or demonstrate antigen-specific naive T cell priming within that compartment.

### 2.4 Step 4: intratumoral restimulation and effector output

Primed effector or memory T cells must return to the tumor, encounter cognate peptide and MHC complexes, and receive signals that preserve effector function. Dendritic cells can contribute to this local phase as well as to lymph node priming. Experimental evidence indicates that CCR7-positive activated dendritic cells can support both naive cytotoxic T cell activation in draining lymph nodes and effector T cell function within tumors (19). In mouse tumors, loss of intratumoral Batf3-dependent dendritic cells also reduced CXCL9 and CXCL10 production and impaired effector T cell trafficking (23). The presence of dendritic cells in a tumor section therefore cannot reveal which of these functions occurred. Spatial organization may be equally important. CCR7-positive dendritic cell niches have been associated with outcome in HNSCC (12), while perivascular regulatory interactions can restrain their stimulatory program in other tumor settings (24). Tumor infiltration, local restimulation, and *de novo* priming must consequently remain separate analytical endpoints.

### 2.5 The missing connections define the central evidence gap

No study identified in the current LSCC evidence set measures the entire sequence in the same patients. The unobserved links include cell-resolved cGAS and STING activation, the repertoire of tumor-derived peptides bound to MHC molecules, dendritic cell maturation and CCR7-dependent trafficking, antigen-specific T cell expansion in tumor-draining lymph nodes, and subsequent clonotype entry into the tumor. Existing LSCC reports instead capture individual nodes through immunohistochemistry, flow cytometry, transcriptomics, or spatial association. Compartment-resolved analysis avoids an unsupported causal interpretation and identifies the links that require direct testing. Evidence within the tumor cell compartment should be linked to antigen-presenting cell state, lymphatic migration, lymph node priming, and intratumoral restimulation using paired samples and functional readouts. Under this framework, “failure to prime” remains one possible location of immune disconnection rather than a diagnosis inferred from low T cell density or a single molecular marker. **Figure 1** summarizes this compartment-resolved framework.

**Figure 1:**
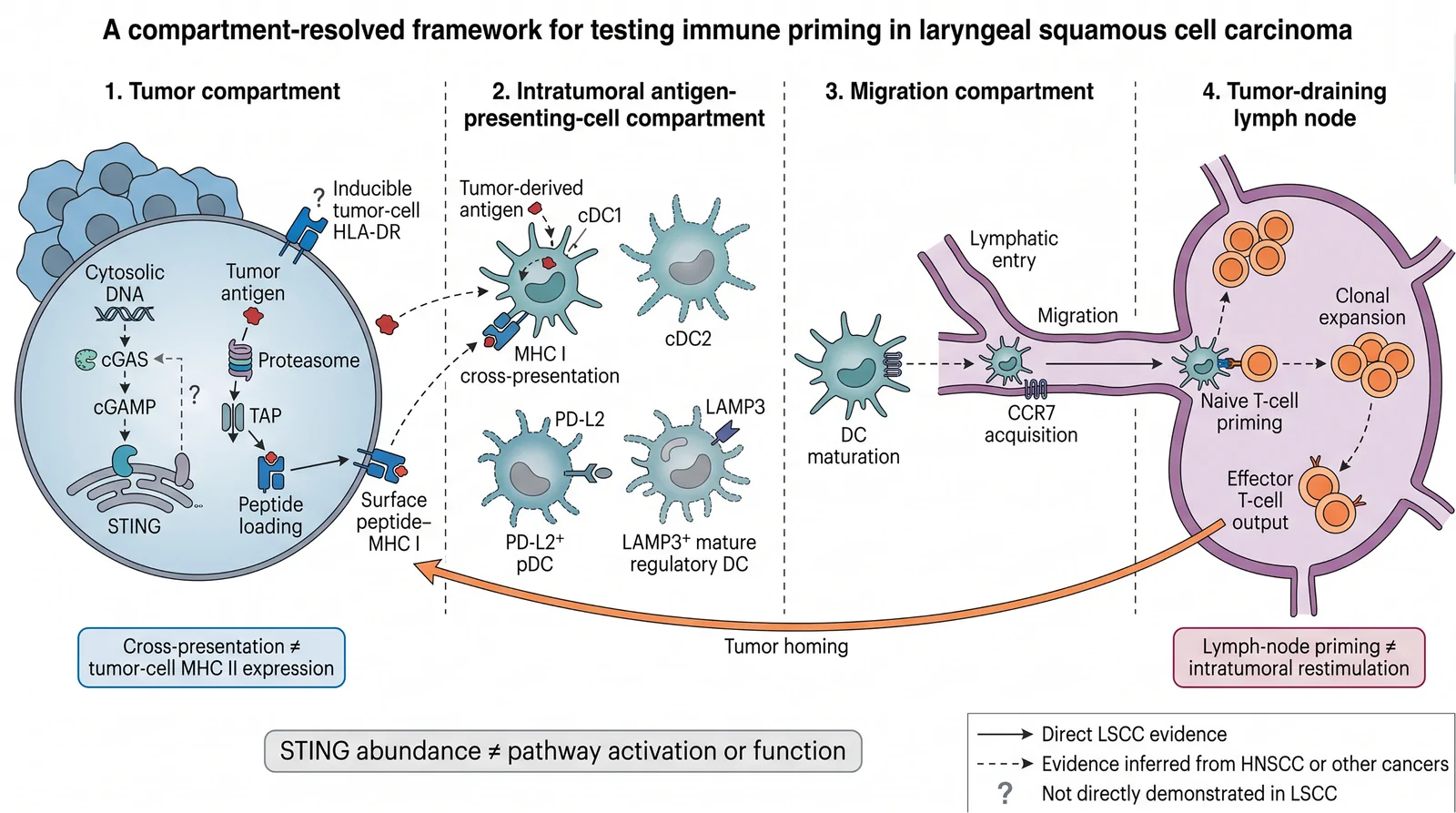
A compartment-resolved framework for testing immune priming in laryngeal squamous cell carcinoma.

The proposed framework follows four anatomical and functional compartments. The tumor cell compartment contains cytosolic DNA sensing through cGAS and STING, MHC class I antigen-processing machinery, and inducible tumor cell HLA-DR. The intratumoral antigen-presenting cell compartment contains cDC1, cDC2, PD-L2-positive plasmacytoid dendritic cells, and LAMP3-positive or mature regulatory dendritic cell states. The migration compartment contains dendritic cell maturation, CCR7 acquisition, and lymphatic entry. The tumor-draining lymph node compartment contains naive T cell priming, clonal expansion, and effector-cell output. Solid outlines indicate nodes supported by direct LSCC observations. Dashed outlines indicate mechanisms inferred from HNSCC or other tumor systems. Question marks indicate connections not directly demonstrated in LSCC. The diagram distinguishes MHC class I cross-presentation from tumor cell MHC class II expression, lymph node priming from intratumoral restimulation, and STING abundance from pathway activation and function.

## 3 Tumor-intrinsic antigen-processing and HLA programs

### 3.1 MHC class I and the antigen-processing machinery

The strongest direct evidence for a presentation defect in LSCC concerns the MHC class I pathway, although it derives from some of the oldest studies included in this Review. In a retrospective immunohistochemical study of 63 primary LSCC lesions, Ogino and colleagues reported frequent downregulation of HLA class I together with LMP2, TAP1, and tapasin (3). Expression of several antigen-processing machinery components correlated with HLA class I abundance and the extent of CD8-positive T cell infiltration. Reduced LMP2 and HLA class I expression, as well as low CD8-positive T cell infiltration, were associated with shorter survival. HLA class I downregulation remained an unfavorable prognostic factor in the reported multivariable analysis (3). A later study of 51 specimens similarly identified reduced or absent HLA class I, TAP1, and LMP7 expression (4).

Together, these studies support recurrent disruption of the MHC class I presentation apparatus in LSCC. They do not determine whether the defects arose through genomic loss, epigenetic repression, altered interferon responsiveness, post-transcriptional regulation, or sampling of spatially heterogeneous tumor regions. Immunohistochemical abundance also cannot reveal which peptides reach the cell surface or whether the displayed repertoire contains immunogenic tumor antigens. The current LSCC evidence set lacks a contemporary, multicenter assessment that jointly measures classical HLA-A, HLA-B, and HLA-C, beta-2-microglobulin, TAP1 and TAP2, tapasin, immunoproteasome components, and the transcriptional regulator NLRC5. It also lacks studies that combine HLA class I immunopeptidomics with antigen-specific T cell testing. This gap is clinically relevant because a tumor can retain detectable HLA class I while presenting an altered or quantitatively insufficient peptide repertoire. Conversely, partial loss on immunohistochemistry does not establish complete resistance to every CD8-positive T cell clone.

Recent HNSCC models indicate that at least some tumor MHC class I programs may be reversible. LSD1 inhibition increased tumor cell MHC class I expression and was linked to dendritic cell recruitment and T cell activation in mouse, humanized, and coculture systems (25). This work provides a mechanistic precedent for epigenetic restoration of antigen visibility. It does not establish the prevalence of LSD1-dependent repression in LSCC or justify clinical use without laryngeal-site validation. For LSCC, the immediate priority is to distinguish irreversible lesions, such as loss of essential pathway genes, from inducible defects that could respond to interferon signaling or epigenetic intervention.

### 3.2 Tumor HLA-DR has at least two plausible meanings

The LSCC HLA-DR literature points in a different direction from the MHC class I literature. In a 56-patient retrospective series, tumor cell HLA-DR was detected in 18 tumors and was associated with well-differentiated histology and favorable disease-specific and relapse-free survival (5). The survival estimates were based on few events, and the reported confidence interval for one hazard estimate was extremely wide. The direction of association is therefore notable, but its precision and generalizability are limited. A subsequent study using tissue imaging, gene-expression analysis, and flow cytometry found that high HLA-DR expression in tumor cells was associated with a lower risk of recurrence, better survival, and enrichment of CD4-positive tumor-infiltrating lymphocytes (6). HLA-DR-positive tumor cells also showed greater PD-L1 and PD-L2 expression (6). The latter observation is compatible with coordinated interferon exposure and illustrates why an antigen-presentation marker can coexist with inhibitory ligands.

These findings support two non-exclusive interpretations. First, tumor HLA-DR may be a surrogate for an interferon-rich, differentiated, or generally inflamed phenotype. Under this model, HLA-DR identifies tumors in which immune recognition has already occurred but is not itself the principal cause of favorable outcome. Second, tumor HLA-DR may contribute functionally to MHC class II antigen presentation and local CD4-positive T cell recognition. Mixed-site HNSCC studies make the second interpretation plausible. A malignant cell interferon and MHC class II program was associated with pembrolizumab response in a 16-patient single-cell cohort and was supported by protein-level and independent bulk-transcriptomic analyses (7). Another HNSCC study linked tumor-specific MHC class II to T cell recruitment, Th1-associated immunity, and response to neoadjuvant immunotherapy (26). Neither study can validate an LSCC-specific biomarker because the anatomical composition, treatment context, and underlying biology of broader HNSCC cohorts are not interchangeable with laryngeal cancer.

The distinction between prognostic and predictive value is unresolved. The title and conclusion of the study by Heng et al. used predictive terminology (6). However, the reported clinical cohort comprised patients treated initially with surgery. It did not include an LSCC cohort treated with anti-PD-1 therapy, nor did it test an interaction between treatment and biomarker status. The study therefore supports a prognostic and immune-context association, not validated prediction of checkpoint inhibitor benefit. Tumor HLA-DR should be described as a hypothesis-generating stratification marker until a prespecified assay, treatment context, and external validation cohort are available.

### 3.3 HLA-DR expression is not equivalent to professional antigen presentation

Productive MHC class II presentation depends on more than surface HLA-DR. A functionally interpretable tumor cell program would require coordinated assessment of CIITA, the invariant chain CD74, HLA-DM-dependent peptide exchange, the resulting peptide repertoire, and recognition by cognate CD4-positive T cells. Priming naive T cells further requires an antigen-presenting context that supplies adequate costimulation and polarization signals. Dendritic cells are specialized for this task, whereas tumor cells may lack or variably express the necessary costimulatory machinery (27, 28). Tumor cell HLA-DR may consequently support intratumoral restimulation of previously primed CD4-positive T cells without initiating *de novo* priming. It may also display peptides that fail to generate a productive response. Static HLA-DR staining cannot distinguish among these possibilities.

### 3.4 Functional criteria for interpreting LSCC antigen presentation

Expression-based observations become mechanistically informative only when they are linked to antigen identity and T cell function. In LSCC, the central unresolved distinction is whether reduced antigen-processing machinery or altered HLA expression changes the surface peptide repertoire sufficiently to impair cognate recognition. HLA-typed MHC class I and MHC class II immunopeptidomics, defined-antigen T cell assays, and autologous tumor-immune cocultures provide complementary ways to test this question. Perturbation of beta-2-microglobulin, CIITA, TAP1, or related pathway components can distinguish causal presentation defects from correlated inflammatory states, whereas rescue experiments can test whether a defect is reversible. Paired tumor and tumor-draining lymph-node material would further help separate local restimulation from *de novo* priming.

For biomarker translation, the strongest evidence would come from prospectively defined and uniformly treated cohorts. Assay platform, cell compartment, scoring threshold, treatment context, and clinical endpoint require prespecification, followed by independent validation. HLA-DR, antigen-processing machinery markers, and related variables should therefore be interpreted as stratification hypotheses until they add reproducible information beyond established clinical and immune covariates. Current evidence indicates that tumor HLA and antigen-processing programs are clinically informative, but it does not establish that they generate productive antigen-specific T cell responses.

## 4 Dendritic cell lineages, states, and migration

### 4.1 Lineage, state, location, and assay should not be collapsed

Interpretation of dendritic cells in LSCC is limited by changing nomenclature and non-equivalent measurement platforms. Ontogeny-based classification separates conventional dendritic cell lineages from plasmacytoid dendritic cells, monocytes, and macrophages, while tissue location and activation programs add further layers of identity (29). In practical tumor studies, cDC1 and cDC2 describe lineage-associated programs. By contrast, CCR7-positive activated dendritic cells and LAMP3-positive or mature regulatory dendritic cells largely describe convergent states that can be acquired by more than one conventional dendritic cell lineage (19, 20, 30). Flow cytometry, immunohistochemistry, and single-cell RNA sequencing capture different aspects of this biology. A flow panel may resolve several surface-defined populations but lose spatial context. Immunohistochemistry preserves spatial context but usually relies on a limited marker set. Transcriptomic clustering can identify programs that are invisible to single-marker staining, yet cluster labels depend on sampling, preprocessing, reference mapping, and annotation choices. Mixed-site HNSCC single-cell atlases further show that viral or carcinogen context can accompany distinct immune states and predicted cell interactions, but those computational relationships do not establish dendritic cell function in LSCC (31, 32).

Here, cross-platform labels are used operationally and are not considered equivalent. CLEC9A, XCR1, and BATF3 support a cDC1 assignment when assessed as a coherent program (29). CD1c and FCER1A support a cDC2-like assignment but do not exclude other dendritic or myeloid states (29). CD123 alone is insufficient to identify a pDC without exclusion markers and a broader pDC program (28, 30). LAMP3 and CCR7 indicate maturation or migration-associated states rather than a unique developmental origin (19, 20). CD1a can occur in several dendritic populations and cannot independently define cDC1, immaturity, or tolerogenic function (29). These limitations are summarized in **Table 1** and should remain visible whenever older LSCC studies are compared with modern single-cell datasets.

**Table 1:** Operational cross-platform definitions of dendritic cell lineages and states used in this review.

Operational entity	Suggested scRNA or spatial markers	Suggested flow or protein definition	Biological role relevant to this review	LSCC evidence status	Critical limitation
cDC1	CLEC9A, XCR1, BATF3, IRF8, CADM1, THBD	Lineage-negative, HLA-DR-positive, CD11c-positive, CD141-high, CD1c-negative, with XCR1 or CLEC9A when feasible	Cross-presentation and CD8 T cell priming	Direct cDC1 abundance and function have not been established in LSCC	CD141 alone is insufficient and can vary with activation. Do not infer cross-presentation from cluster labels.
cDC2	CD1C, CLEC10A, FCER1A, IRF4, HLA-DRA	Lineage-negative, HLA-DR-positive, CD11c-positive, CD1c-positive, CD141-low	MHC-II presentation and CD4 T cell priming with context-dependent cross-presentation	LSCC studies describe conventional DC subsets but do not provide a validated cDC2 functional map	CD1c can be expressed by inflammatory myeloid cells. Exclude monocytes with an explicit panel.
pDC	GZMB, JCHAIN, TCF4, IL3RA, CLEC4C, IRF7	Lineage-negative, HLA-DR-positive, CD123-high, CD303-positive or CD304-positive, CD11c-low	Type I interferon production with tolerogenic or suppressive states in tumors	PD-L2-positive tumor-infiltrating pDCs are directly reported in LSCC	CD123 alone is not specific. The LSCC evidence is associative and does not prove failed priming.
LAMP3-positive or mregDC state	LAMP3, CCR7, FSCN1, CCL19, CD274, PDCD1LG2, MARCKSL1	No universal surface-only panel. Use activation and migration markers with lineage assignment or trajectory analysis	Mature migratory state with coexisting immunostimulatory and regulatory programs	LAMP3-positive DCs are reported in laryngeal precancer and LSCC-related single-cell studies	A state rather than a single lineage. It may arise from cDC1 or cDC2 and does not prove successful migration to tDLNs.
CD1a-positive DC-like cells	CD1A with context-dependent FCER1A or CD207	CD1a-positive, HLA-DR-positive cells with exclusion of epithelial and lymphoid cells	Immature tissue DC or Langerhans-like phenotype depending on site	Older LSCC histology links CD1a-positive infiltration with HBD-2 expression	CD1a is not equivalent to cDC1 and is insufficient for lineage or function.
Monocyte-derived DC generated ex vivo	CD83, CD80, CD86, HLA-DRA after culture with GM-CSF and IL-4	Culture-defined CD14-derived cells with maturation markers	Experimental antigen loading and T cell stimulation	Multiple older LSCC studies measured generated MoDC phenotype and function	These cells are culture products and should not be mapped directly onto intratumoral DC states.
Follicular dendritic cell	CXCL13-associated follicular markers, CR2, CR1, FDCSP	Non-hematopoietic stromal network in follicles or TLS	Antigen retention and B-cell organization rather than conventional T cell priming	Occasional laryngeal literature mentions follicular DCs	Do not combine follicular DCs with cDC1, cDC2, pDC, or monocyte-derived DCs.

### 4.2 cDC1 and the unmeasured cross-presentation axis

cDC1s provide the clearest mechanistic bridge between tumor antigen uptake and CD8-positive T cell cross-priming. In experimental cancer systems, they capture cell-associated material, route exogenous antigen into MHC class I presentation, receive activating or CD4-positive T cell licensing signals, and support tumor-specific cytotoxic T cell responses (17, 18, 27). Their recruitment can depend on chemokines produced by natural killer cells, and their abundance has been associated with T cell-inflamed tumors across cancer types (33). These mechanisms justify measuring cDC1s in LSCC, although a quantitative or functional cDC1 deficit remains unproven. The current LSCC literature set contains no study that combines a lineage-resolving cDC1 panel with a cross-presentation assay using a defined antigen. CLEC9A, XCR1, BATF3, and appropriate exclusion markers should therefore be treated as a proposed measurement set. They should not be presented as an established LSCC signature.

Cell number alone would still be inadequate. Antigen uptake, maturation, peptide loading, cytokine production, CCR7-dependent migration, and T cell priming are separable functions. Experimental studies of DNGR-1 signaling and the epigenetic regulator CARM1 show that receptor signaling or chromatin state can alter cross-presentation without being reducible to dendritic cell abundance (34, 35). A tumor may contain cDC1-like cells that remain poorly activated or acquire regulatory programs after antigen uptake. Conversely, a low cDC1 count in one tumor region may not represent the draining lymph node or the entire lesion. Functional testing should combine phenotyping with antigen-specific coculture, migratory assays, and paired tumor and lymph node analysis.

### 4.3 cDC2 and CD4-positive T cell immunity

cDC2s are relevant to the HLA-DR findings because they can prime CD4-positive T cells, whereas HLA-DR-positive tumor cells are more plausibly positioned to participate in local recognition or restimulation. These processes should not be merged. Recent mechanistic work also indicates that activated states can arise from cDC1s or cDC2s, and that cDC2-derived activated cells may support CD8-positive T cell responses through context-dependent cross-dressing (19). In pancreatic tumor models, activated cDC2s carrying tumor antigen were required for draining-node CD4-positive Th1-cell priming under combination immunotherapy (36). Experimental immune-complex models further show that cDC2s can cross-present under defined conditions (37). In HNSCC, mature tertiary lymphoid structures contained DC-LAMP-positive dendritic cells spatially associated with progenitor-exhausted CD4-positive and activated CD8-positive T cell responses (38). These findings challenge a model restricted to cDC1, but none has demonstrated an LSCC-specific priming route. Direct LSCC data on cDC2 abundance, maturation, antigen repertoire, and tumor-specific CD4-positive T cell priming remain sparse in the current evidence set.

### 4.4 What older LSCC dendritic cell studies can and cannot show

Earlier LSCC studies remain informative when their assay boundaries are preserved. An immunohistochemical study of 34 LSCC specimens examined human beta-defensin 2 and CD1a-positive infiltrating cells, with a smaller subset used for RNA analysis (39). The report supports an association between the epithelial antimicrobial peptide and a broadly defined CD1a-positive population. It does not directly demonstrate chemotaxis, dendritic cell activation, lineage identity, or antigen-specific priming. Two studies using peripheral blood and ex vivo assays addressed dendritic cell competence from a different perspective. Tumor-lysate-pulsed CD83-positive monocyte-derived dendritic cells generated from 60 patients with laryngeal cancer expressed higher levels of B7-H1, B7-H4, CD200, and CD200R than cells from healthy donors, with expression varying by tumor grade (40). Cells generated *in vitro* are not equivalent to intratumoral cDC1, cDC2, or pDC populations. A longitudinal study of 46 patients reported impaired circulating myeloid dendritic cell counts and monocyte-derived dendritic cell phenotype or stimulatory capacity before treatment, followed by partial recovery after surgery with or without radiotherapy (41). The mixed leukocyte reaction measured general T cell stimulatory competence rather than tumor antigen-specific priming. Together, these studies suggest systemic and inducible dendritic cell abnormalities in patients with LSCC, but they cannot locate the defect within the pathway from tumor antigen acquisition to lymph node priming.

### 4.5 PD-L2-positive pDCs represent an association, not yet a mechanism

The most direct contemporary LSCC dendritic cell study concerns pDCs. Ji and colleagues compared dendritic and T cell subsets in tumor tissue, adjacent non-tumor tissue, and peripheral blood, with tissue imaging used to assess pDC and T cell localization (8). Tumor-infiltrating pDCs were increased relative to adjacent tissue, were more abundant in node-positive disease, and were associated with unfavorable outcome. Their abundance correlated negatively with CD8-positive T cell infiltration. The study reported a higher proportion of PD-L2-positive pDCs in tumor than in paired adjacent non-tumor tissue, whereas the proportion of PD-L1-positive pDCs did not differ between those tissues (8). These findings identify pDC abundance and phenotype as clinically relevant correlates. They do not demonstrate that pDC-derived PD-L2 caused the reduction in CD8-positive cells, that the interaction occurred through PD-1 *in situ*, or that pDCs impaired priming in draining lymph nodes.

pDCs are also not defined solely by inhibitory ligands. Their functions depend on activation state and can include type I interferon production, immune regulation, and limited antigen-presentation activity (28, 30). The key LSCC questions are therefore functional and spatial. Future studies should determine the location of PD-L2-positive pDCs, whether within tumor nests, stroma, lymphatic interfaces, or draining lymph nodes. These studies should also test whether the cells retain type I interferon competence and whether their abundance relates to tumor-specific T cell clonotypes. Because PD-1 blockade already interrupts signaling from both PD-L1 and PD-L2 through PD-1, the existing data do not justify routinely combining separate PD-L2 and PD-1 blockade. pDC state and location are stronger immediate candidates for biological stratification than for a clinical treatment recommendation.

### 4.6 LAMP3-positive and mature regulatory states combine activation with restraint

Single-cell studies have shifted attention from dendritic cell quantity to state. A single-cell and spatial study of laryngeal tissues from 36 patients found that vocal cord leukoplakia already shared immunoregulatory features with LSCC (9). A separate analysis reported abundant LAMP3-positive dendritic cells in leukoplakia and positive correlations with regulatory T cells and exhausted CD8-positive T cell states (10). This pattern is consistent with early dendritic cell remodeling, but it neither establishes that LAMP3-positive cells caused immune escape nor proves that they migrated to lymph nodes.

Cross-tumor atlases explain why LAMP3 requires cautious interpretation. The mature regulatory dendritic cell program can be induced in cDC1s and cDC2s after tumor antigen uptake and includes maturation genes together with immunoregulatory molecules (20). Larger pan-cancer analyses suggest that LAMP3-positive populations can have multiple cellular origins and pleiotropic functional programs (42). Recent functional work similarly identifies CCR7-positive activated states derived from multiple conventional dendritic cell lineages that coexpress stimulatory and inhibitory molecules (19). LAMP3 positivity may therefore indicate antigen experience and maturation, regulatory adaptation, migratory potential, or a combination of these features. Assignment of a uniformly suppressive function from one transcript or antibody stain is not defensible.

### 4.7 CCR7 migration and the tumor-draining lymph node remain the missing bridge

It remains unknown whether LSCC dendritic cells transport tumor antigens to draining cervical lymph nodes and initiate tumor-specific T cell responses. In melanoma models, CCR7-dependent trafficking by tumor antigen-bearing dendritic cells was required for lymph node priming (21). Expansion and activation of intratumoral CD103-positive dendritic cell progenitors enhanced antigen transport and responses to checkpoint blockade (43). As noted in Section 2.4, CCR7-positive activated dendritic cells are required for both lymph node priming and intratumoral effector maintenance (19). In mixed-site HNSCC, the spatial organization of CCR7-positive dendritic cell niches was associated with survival in patients sampled before pembrolizumab treatment (12). Cross-tumor spatial analyses further suggest that perivascular CCR7-positive dendritic cell niches can support stimulation while remaining vulnerable to regulatory T cell suppression (24). None of these findings directly demonstrates LSCC-specific migration or priming.

The most informative LSCC validation setting would pair the primary tumor with anatomically mapped draining lymph nodes while distinguishing uninvolved, metastatic, and treatment-exposed nodes. Harmonized flow cytometry, spatial protein profiling, and single-cell transcriptomics could resolve dendritic-cell lineage and state, while peptide presentation, validated antigen tracing, T cell receptor clonotypes, and antigen reactivity could connect tumor and lymph-node compartments. These approaches define the evidence needed to test the missing bridge rather than implying that failed migration or failed priming has already been demonstrated. At present, LSCC studies support altered dendritic-cell abundance and state at several nodes, but not a continuous causal pathway.

## 5 cGAS-STING signaling across expression, activation, output, and function

### 5.1 STING biology must be resolved by cellular compartment

The cGAS-STING pathway can connect genomic or mitochondrial stress to innate immunity, but its consequence depends on where the signal originates and which cell receives it. Tumor cell cGAS can generate cyclic GMP-AMP that is transferred to dendritic cells or macrophages in experimental systems, allowing host cells to produce type I interferon even when tumor cell STING output is limited (44). Intrinsic cGAS-STING activity in dendritic cells can be constrained by cellular metabolism and can influence conventional dendritic cell function (45). Other models show transfer of tumor-derived cyclic GMP-AMP to endothelial cells, with effects on vascular state and lymphocyte entry (46). These intercellular routes are biologically plausible but have not been demonstrated in LSCC. Tumor cells, dendritic cells, macrophages, endothelial cells, and fibroblasts should therefore be analyzed separately rather than combined into one tissue-level STING score.

### 5.2 Direct LSCC evidence measures abundance, not pathway competence

The principal direct LSCC study evaluated 59 tumors without distant metastasis, classified as stage I through IV by immunohistochemistry for STING, cGAS, CD8, CD68, and CD163 (11). Higher tumor cell STING expression was associated with stage IV disease, advanced primary tumor category, higher histological grade, and lymph node positivity. Its association with stage IV disease remained after adjustment for selected clinical variables and cGAS expression (11). This study establishes a clinicopathological association with tumor cell protein abundance. It does not establish recent STING activation, downstream interferon signaling, a causal role in progression, dendritic cell cross-priming, or benefit from a STING agonist. The conclusion that high expression might identify a therapeutic opportunity is a hypothesis that requires functional testing, not a validated predictive biomarker.

### 5.3 A four-layer framework for interpreting STING

STING should be assessed across four linked but non-equivalent layers. Expression can be assessed using cell-resolved cGAS and STING transcript or protein abundance. Activation requires biochemical evidence such as phosphorylation of STING, TBK1, and IRF3, together with IRF3 nuclear localization and cyclic GMP-AMP quantification when technically feasible. Output should be evaluated by measuring type I interferons, CXCL10, other interferon-stimulated genes, and context-dependent NF-κB-associated mediators (15, 16). Functional assessment should determine whether signaling promotes dendritic cell maturation, CCR7-dependent migration, cross-presentation, antigen-specific T cell expansion, and tumor control. Measurement at one layer cannot substitute for the next. A valid LSCC activation index must also preserve cell type and spatial location because tumor cell and host cell signaling can have different or even opposing consequences. **Figure 2** summarizes this four-layer framework and indicates the connections that remain untested in LSCC.

**Figure 2:**
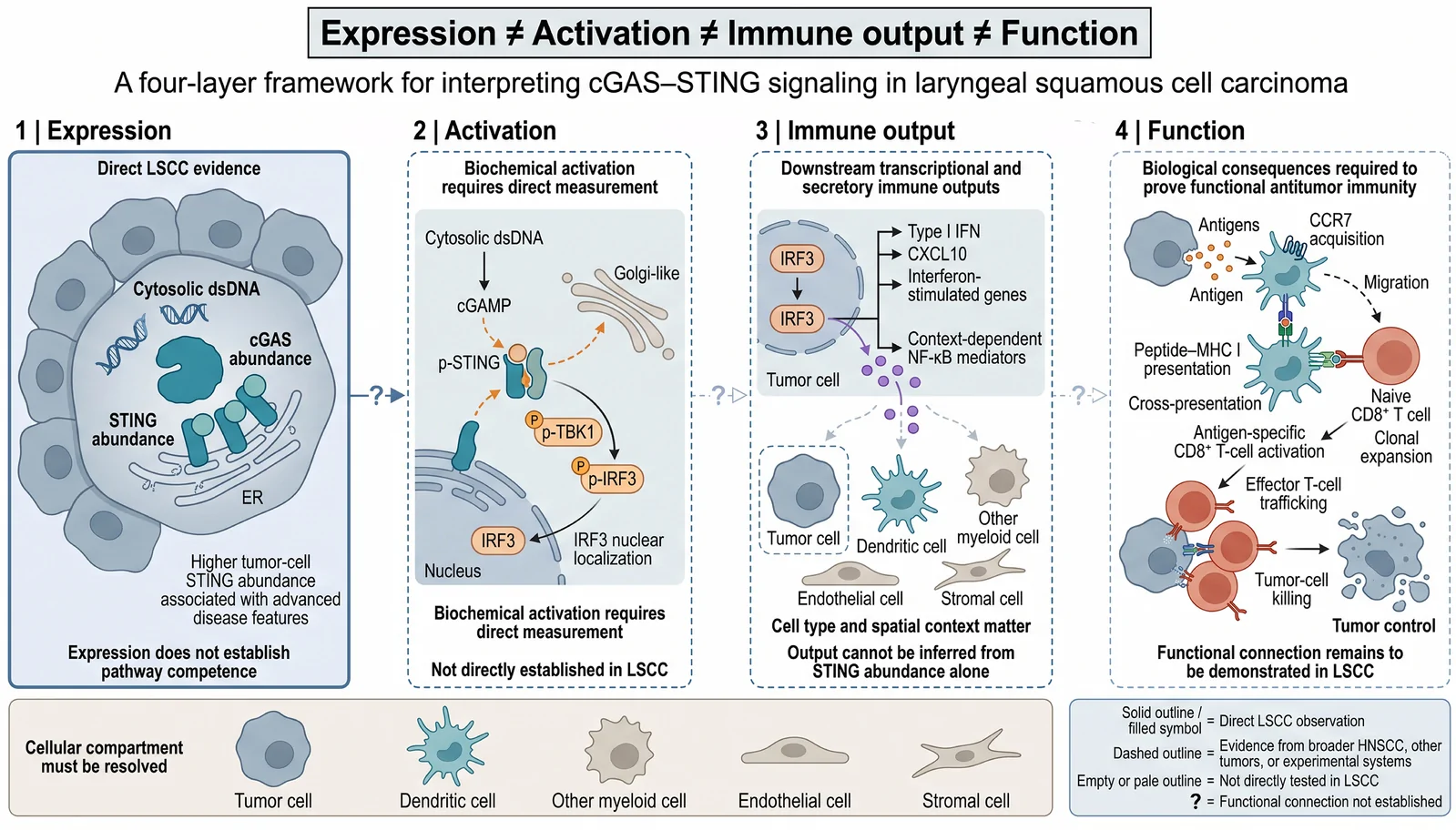
A four-layer framework for interpreting cGAS-STING signaling in laryngeal squamous cell carcinoma.

Expression of cGAS or STING is separated from biochemical activation, downstream immune output, and functional consequences. Each layer is resolved across tumor cells, dendritic cells, other myeloid cells, endothelial cells, and stromal cells. The current direct LSCC evidence primarily occupies the tumor cell expression layer. Activation requires measurements such as phosphorylated TBK1 or IRF3 and IRF3 nuclear localization. Output includes type I interferon, CXCL10, and interferon-stimulated gene programs (15, 16). Function requires evidence of dendritic cell maturation or migration, cross-presentation, antigen-specific T cell responses, or tumor control. Solid symbols identify direct LSCC observations. Dashed symbols identify HNSCC or cross-tumor evidence. Empty cells identify untested LSCC layers.

### 5.4 Why high tumor cell STING can accompany advanced LSCC

TRIM29 provides another candidate mechanism for uncoupling STING abundance from downstream output. A recent review summarized context-dependent roles for TRIM29 in cancer progression, including evidence from squamous cell carcinoma models (77). The direction of effect is not uniform across squamous malignancies: tumor-promoting phenotypes have been reported in lung squamous cell carcinoma, whereas tumor-suppressive associations have been described in esophageal squamous cell carcinoma (77). In DNA-virus models, TRIM29 induced K48-linked ubiquitination and degradation of STING, thereby limiting type I interferon production (78). In a viral myocarditis model, TRIM29 stabilized PERK through SUMOylation, increased endoplasmic reticulum stress-associated reactive oxygen species, promoted TBK1 oxidation, impaired TBK1 dimerization and downstream TBK1-IRF3 activation, and reduced type I interferon output (79). These studies make TRIM29 mechanistically relevant to an expression-output disconnect, but their disease contexts are distinct from LSCC and none establishes TRIM29-dependent immune escape in LSCC.

The association between high tumor cell STING abundance and advanced LSCC is not necessarily paradoxical. High STING abundance may reflect reactive upregulation during genomic stress without effective downstream signaling. The pathway may be intact proximally but interrupted at TBK1, IRF3, interferon, or antigen-presenting cell stages. Chronic or spatially restricted signaling may differ from acute activation. Tumor cell signaling may have different consequences from dendritic cell or stromal signaling. Finally, STING abundance may be a companion marker of tumor evolution rather than a driver of progression. Cross-tumor studies of SMARCAL1 and TREX1 illustrate how DNA damage control or cytosolic DNA clearance can restrain tumor-intrinsic cGAS-STING signaling while reshaping checkpoint or immune programs (47, 48). None of these alternatives, including a TRIM29-mediated block, has been tested directly in LSCC.

Broader HNSCC findings reinforce the need for contextual interpretation. In one HNSCC analysis, higher pathway scores were observed in immunotherapy responders, and accompanying experiments linked STING signaling to macrophage polarization (49). Another study linked NOVA1 to STING phosphorylation and chemokine-related outputs in HNSCC cells and clinical tissues (50). Unlike the association with advanced stage observed in LSCC, compartment-resolved STING abundance was associated with favorable disease control in two radiotherapy-treated HNSCC cohorts (51). One of these cohorts was oropharyngeal and strongly influenced by p16 status. These studies are not contradictory once anatomical site, treatment, viral context, cell compartment, and assay layer are considered. They also cannot be pooled into a single LSCC direction of effect.

### 5.5 Therapeutic activation requires a functional diagnosis

Preclinical HNSCC studies provide several routes by which DNA damage, ferroptosis, engineered delivery, or relief of transcriptional repression can engage STING-associated immunity (52–55). Cross-tumor work with manganese links cGAS-STING sensitization to dendritic cell maturation and adaptive immune responses (56). Its clinical component was an open-label phase I cohort of 22 patients with heterogeneous advanced solid tumors who received combination therapy, not an LSCC efficacy study (56). Such results establish experimental tractability, but they do not show that an LSCC with high baseline STING staining will respond to further agonism. Human experience also argues against expression-only selection. In a phase Ib study involving patients with advanced solid tumors or lymphomas, intratumoral MIW815 combined with PD-1 blockade produced pharmacodynamic activity but limited antitumor responses (57). More recent ulevostinag studies showed target engagement, while the randomized HNSCC component included only 18 participants and was insufficient for an LSCC-specific conclusion (58).

For LSCC, STING-directed translation depends on a functional diagnosis rather than on expression alone. The most informative evidence would establish pathway components, activation competence, cell-resolved downstream output, and a connection to dendritic-cell maturation and antigen-specific T cells. Ex vivo agonist challenge, pathway blockade, or rescue can distinguish responsive from non-responsive states and identify the compartment responsible for the effect. Longitudinal sampling can determine whether signaling is transient or sustained. High STING expression alone is therefore not an adequate enrollment biomarker. A therapeutic rationale exists only when the pathway produces the required immune output in the relevant cell and time window and when that output supports antigen presentation and priming.

A preclinical study identified SB24011, a small molecule that disrupts the STING-TRIM29 interaction and blocks TRIM29-induced STING degradation, thereby increasing STING protein abundance and potentiating the activity of a STING agonist and anti-PD-1 therapy (80). This provides proof of concept for pharmacologically relieving TRIM29-mediated restraint, but it does not establish efficacy in LSCC. TRIM29-directed translation in LSCC would require evidence of TRIM29 expression in the relevant cellular compartment, demonstrable TRIM29-dependent STING turnover or suppression of TBK1/IRF3 output, and functional immune rescue after perturbation.

## 6 Temporal, etiologic, genetic, and population modifiers of priming

### 6.1 Immune remodeling may precede invasion, but its sequence is unresolved

Laryngeal carcinogenesis provides an opportunity to ask whether presentation and dendritic cell abnormalities arise before invasive cancer. Earlier synthesis implicated infiltrating immune and inflammatory cells in progression from dysplasia to invasive disease, but did not establish an antigen-specific priming mechanism (59). Several recent datasets indicate that immune remodeling is detectable in premalignant lesions. A targeted genomic and immune analysis compared 15 dysplasias that progressed with 31 that did not and associated higher lymphocyte infiltration and selected immune transcripts with malignant progression (60). A separate 110-patient dysplasia cohort found that stromal immune features, histological grade, and exposure variables were associated with progression (61). However, the reported immune effect sizes were modest, and the direction of some associations was inconsistent with a straightforward protective interpretation. Transcriptomic profiling also separated laryngeal dysplasias by immune-related expression patterns associated with clinical behavior, although the study did not measure antigen-specific priming (62). Single-cell studies identified evolving epithelial, stromal, and mononuclear-phagocyte programs across leukoplakia and early carcinoma (63), while a 36-patient single-cell and spatial atlas described vocal cord leukoplakia as a hybrid state sharing immunoregulatory features with LSCC (9). LAMP3-positive dendritic cells and exhausted T cell-associated states were also reported in leukoplakia in an independent analysis (10).

Immune remodeling is detectable in premalignant lesions, although whether it precedes or follows epithelial injury remains unresolved. High T cell density in a progressing lesion may reflect effective surveillance, chronic antigen exposure without clearance, or inflammation associated with tissue damage. Cross-sectional comparisons cannot determine the temporal or causal direction. Longitudinal sampling of the same lesion, paired with antigen-specific and functional measurements, is required to distinguish a causal bottleneck from an accompanying immune state.

### 6.2 HPV, carcinogen exposure, and treatment alter the context

Etiologic variables should be treated as modifiers rather than assumed explanations. Tobacco and alcohol are major risk factors for laryngeal dysplasia, whereas the causal contribution of HPV remains uncertain (64). LSCC studies should distinguish p16 staining, HPV DNA detection, and transcriptionally active viral infection rather than merge them into one binary variable. Tobacco-associated genomic injury may increase the substrate for innate DNA sensing or neoantigen formation, but it does not guarantee cytosolic DNA accumulation, STING activation, peptide presentation, or successful priming. Exposure history should therefore be measured alongside pathway function.

Treatment history creates another source of heterogeneity. In recurrent or metastatic HNSCC, the number of prior treatment lines was strongly associated with changes in lymphocyte abundance (2). Radiation and systemic therapy can release antigen and alter innate signals, yet their effects depend on dose, timing, tissue compartment, and host cell state. Experimental evidence that radiotherapy can induce a dendritic cell program that impairs cross-presentation illustrates that treatment-related DNA damage is not uniformly immunogenic (65). This mechanism has not been demonstrated in LSCC and should not be used to predict an individual treatment response.

### 6.3 A temporal study should preserve clinically distinct states

A useful LSCC timeline should separate normal mucosa, leukoplakia or dysplasia, untreated primary tumor, nodal metastasis, and treatment-exposed recurrent disease. It should also preserve distinctions among glottic, supraglottic, and subglottic subsites, because broad HNSCC categories conceal site-specific molecular and immune differences (1). Paired longitudinal specimens are preferable to a pseudotemporal ordering assembled from unrelated patients. Where a stage has no direct evidence, the gap should remain visible rather than be filled by a cross-tumor assumption. **Table 2** summarizes the direct LSCC and laryngeal premalignancy evidence supporting the nodes discussed in Sections 3 to 6 and separates each observation from what it cannot prove.

**Table 2:** Direct LSCC and laryngeal premalignancy evidence relevant to antigen presentation, dendritic cell states, and cGAS-STING signaling.

Study	Year	Disease material	Evidence type	Direct observation relevant to this review	Priming stage addressed	Main limitation
Dynamic tumor microenvironment remodeling from laryngeal leukoplakia to carcinoma revealed by single-cell transcriptomics (63).	2026	Laryngeal leukoplakia and early carcinoma specimens	Single-cell RNA sequencing	Cellular remodeling across leukoplakia grades and carcinoma, with epithelial, fibroblast, and mononuclear-phagocyte changes	Temporal context before invasion	Small cohort and computational associations. It did not test antigen-specific priming.
Immunohistochemical characterization of the tumor immune microenvironment in laryngeal premalignancy: insights into early immune alterations in carcinogenesis (61).	2026	110 excised laryngeal dysplasias	Retrospective IHC and digital pathology cohort	Stromal immune features, dysplasia grade, and exposure variables were associated with progression	Premalignant immune context	Associative markers with modest effect sizes. No DC or antigen-presentation function was measured.
Single-cell Deciphering of the Progression Trajectories of the Tumor Ecosystem in Laryngeal Squamous Cell Carcinoma (10).	2026	LSCC, vocal cord leukoplakia, and precursor datasets	Integrated single-cell and bulk transcriptomics with selected functional validation	LAMP3-positive DCs were abundant in VCL and correlated with Tregs and exhausted CD8 T cells	DC state and premalignant context	Correlation does not establish DC lineage origin, migration, or causal T cell suppression.
Single-cell RNA sequencing reveals hypoxia-associated stress response signatures in the immune and stromal microenvironment of laryngeal squamous cell carcinoma (66).	2026	Immune-enriched LSCC single-cell dataset	Secondary single-cell analysis with THP-1 validation	Hypoxia-associated programs were strongest in myeloid populations and included CXCL8 and stress-response genes	Contextual modifier	Reanalysis of one dataset. THP-1 validation does not establish LSCC DC function or priming.
Comprehensive Single-Cell RNA Atlas of Human Laryngeal Normal, Preneoplastic, and Tumorigenic States (9).	2025	36 patients with polyps, leukoplakia, or LSCC	Single-cell RNA sequencing and spatial transcriptomics	VCL showed a transitional microenvironment with early immunosuppressive features and immunoregulatory-state differences	Temporal and spatial context	State annotations do not directly demonstrate antigen presentation, migration, or lymph node priming.
A Transcriptomic Analysis of Laryngeal Dysplasia (62).	2024	Laryngeal dysplasia specimens	Targeted immune-transcriptomic profiling	Immune-response transcriptional patterns differed between progressive and non-progressive dysplasia	Premalignant immune context	Exploratory transcriptomic classification and limited functional validation.
Increased tumor-infiltrating plasmacytoid dendritic cells express high levels of PD-L2 and affect CD8(+) T lymphocyte infiltration in human laryngeal squamous cell carcinoma (8).	2024	LSCC tumor, adjacent non-tumor tissue, and blood	Flow cytometry, IHC, immunofluorescence, and survival analysis	Tumor-infiltrating pDCs were enriched and associated with nodal disease and poor outcomes. Their abundance was negatively correlated with CD8 TIL density, and the cells expressed PD-L2	Intratumoral DC state	Association and phenotype only. It did not prove pDC-mediated CD8 exclusion or failed priming.
Elevated Tumor Cell-Intrinsic STING Expression in Advanced Laryngeal Cancer (11).	2023	59 non-metastatic laryngeal SCC specimens	IHC and multivariable association analysis	Higher tumor cell STING staining was associated with advanced stage, higher grade, and nodal positivity	Innate sensing expression	Expression was measured without pathway activation, interferon output, or functional agonist response.
High Expression of Tumor HLA-DR Predicts Better Prognosis and Response to Anti-PD-1 Therapy in Laryngeal Squamous Cell Carcinoma (6).	2023	LSCC tissue microarrays and fresh tissues	MHC-II gene analysis, IHC, immunofluorescence, flow cytometry, and outcome analysis	High tumor HLA-DR was associated with better outcome and greater CD4 T cell infiltration	Tumor MHC-II display	HLA-DR expression does not establish professional APC activity or a validated predictive biomarker.
The Genetic and Immunologic Landscape Underlying the Risk of Malignant Progression in Laryngeal Dysplasia (60).	2023	15 progressing and 31 non-progressing laryngeal dysplasias	Targeted sequencing, IHC, and immune-gene profiling	TIL patterns and immune-gene correlations differed by malignant progression	Premalignant immune context	Small retrospective cohort. It did not resolve antigen specificity or causal timing.
European Laryngological Society position paper on laryngeal dysplasia Part I: aetiology and pathological classification (64).	2021	Laryngeal dysplasia literature	European Laryngological Society position paper	Defines dysplasia, risk factors, grading limitations, and uncertainty around HPV causality	Clinical and pathological context	Consensus context rather than direct priming evidence.
Immunohistochemical detection of “ex novo” HLA-DR in tumor cells determines clinical outcome in laryngeal cancer patients (5).	2021	56 primary LSCC specimens	Retrospective IHC and survival analysis	Tumor cell HLA-DR positivity occurred in a subset and was associated with better disease-specific and relapse-free survival	Tumor MHC-II display	Small single-center cohort and limited events. Functional presentation was not tested.
Current understanding of the tumor microenvironment of laryngeal dysplasia and progression to invasive cancer (59).	2016	Laryngeal dysplasia literature	Narrative review	Summarizes immune and inflammatory contributions to progression from dysplasia to carcinoma	Premalignant context	Review-level evidence. It cannot determine sequence or causality.
Expression of selected regulatory molecules on the CD83+ monocyte-derived dendritic cells generated from patients with laryngeal cancer and their clinical significance (40).	2013	60 laryngeal cancer patients and 15 controls	Ex vivo tumor-lysate-pulsed monocyte-derived DC flow cytometry	Regulatory molecules B7-H1, B7-H4, CD200, and CD200R were increased on generated CD83-positive MoDCs	Ex vivo DC phenotype	MoDCs generated *in vitro* are not equivalent to intratumoral cDC lineages.
[Association of antigen processing machinery and HLA class I defects with clinicopathological outcome in laryngeal squamous cell carcinoma] (4).	2012	51 LSCC specimens	IHC of TAP1, LMP7, and HLA class I	Frequent APM component and HLA-I downregulation, with correlated expression and clinicopathological associations	Tumor antigen processing and MHC-I	Archived IHC does not measure peptide repertoire, antigen specificity, or presentation function.
[Expression of human beta-defensin-2 in laryngeal squamous cell carcinoma and its correlation with CD1a(+) dendritic cells infiltrating in tumor] (39).	2009	34 LSCC paraffin specimens with a smaller RNA subset	IHC and *in-situ* hybridization	HBD-2 expression correlated with tumor differentiation and infiltration by CD1a-positive cells	DC recruitment hypothesis	CD1a is not a lineage-specific cDC1 marker. Chemotaxis and activation were not directly tested.
Therapeutic influence on circulating and monocyte-derived dendritic cells in laryngeal squamous cell carcinoma patients (41).	2009	46 LSCC patients and 15 controls	Longitudinal blood DC analysis, generated MoDC phenotype, and mixed leukocyte reaction	Circulating mDC number and MoDC phenotype/function were impaired before treatment and partly recovered after surgery	Systemic and ex vivo DC competence	Peripheral and generated MoDC assays do not establish tumor or lymph node priming.
HLA class I antigen down-regulation in primary laryngeal squamous cell carcinoma lesions as a poor prognostic marker (3).	2006	63 primary laryngeal SCC lesions	IHC of HLA-I and multiple APM components	APM defects accompanied HLA-I downregulation, lower CD8 infiltration, and worse survival	Tumor antigen processing and MHC-I	Protein staining and outcome association do not directly quantify presented peptides or antigen-specific T cell recognition.

### 6.4 Host immunogenetics and population heterogeneity

Host immunogenetic diversity is particularly relevant to an antigen-presentation framework because HLA genotype shapes the repertoire of peptides that can be displayed, and HLA allele frequencies vary substantially across populations (71, 72). The present LSCC literature is not sufficiently large or geographically diverse to determine whether HLA genotype, germline variation, genetic ancestry, or population-associated exposures modify antigen-processing defects, dendritic-cell states, or cGAS-STING competence. These factors should not be collapsed into a single racial variable. Where available, self-reported race or ethnicity, genetically inferred ancestry, HLA genotype, geography, tobacco and alcohol exposure, viral status, laryngeal subsite, and treatment history should be analyzed as distinct but potentially interacting covariates. Large multicenter prospective studies that include diverse populations and harmonized biospecimen processing will be necessary to test whether candidate immune-priming phenotypes generalize across groups and to avoid HLA-restricted biomarkers or therapeutics that perform unevenly because of population-specific allele representation.

## 7 Discussion

### 7.1 Prioritizing actionable nodes within the LSCC immune-priming axis

Target prioritization should reflect both the strength of LSCC evidence and intervention tractability rather than pathway prominence alone. We therefore separate candidates supported by direct LSCC observations from those transferred from broader HNSCC or other tumor systems, and we distinguish antibody-accessible surface molecules from intracellular pathways that require pharmacological or genetic modulation. The hierarchy below defines research priorities for mechanistic and translational validation. It is not a clinical treatment recommendation. Candidates within the same tier are not ranked further because their evidence types and intervention modalities are not directly comparable.

The highest LSCC-specific validation priority includes the MHC-I antigen-processing machinery, PD-L2-positive pDC states, and functional cGAS-STING competence. Recurrent loss of HLA class I and antigen-processing components provides the strongest direct evidence for a potentially actionable presentation bottleneck (3, 4). Restoration strategies may involve reversible transcriptional or epigenetic regulators such as LSD1, but success must be judged by recovered peptide display and cognate T cell recognition rather than by HLA abundance alone (25). PD-L2-positive pDCs are directly associated with nodal disease, adverse outcome, and lower CD8-positive T cell infiltration in LSCC (8). PD-L2 is antibody accessible, but separate PD-L2 blockade cannot yet be prioritized clinically because PD-1 blockade already interrupts PD-L2 signaling and causality remains unproven. STING is similarly a high-priority functional stratification node rather than an expression-selected drug target. Agonism is most defensible when cell-resolved activation, downstream output, and immune rescue are demonstrable (11, 57, 58).

Second-tier candidates are cDC1-directed strategies centered on CLEC9A, XCR1, and FLT3L. CLEC9A and XCR1 represent candidate receptors for cDC1-directed antigen-delivery strategies; however, their antibody-targeting utility and functional relevance have not been validated in LSCC. FLT3L can expand cDC1 populations and strengthen draining-node priming in experimental systems (18, 22, 29), but this strategy also lacks direct functional validation in LSCC. CCR7 is better viewed as a migration-enabling node than as a conventional blockade target because inhibiting CCR7 could impair the trafficking required for priming (19, 21).

Lower-priority exploratory candidates include the LAMP3 or mature regulatory dendritic-cell program and the tumor HLA-DR or CIITA axis. LAMP3 marks a mature state that may arise from several dendritic-cell lineages and is not a sufficiently specific surface target by itself (9, 10, 20). Tumor HLA-DR has reproducible prognostic and immune-context associations in LSCC (5, 6), but its current value is greater as a biomarker or functional readout than as a direct therapeutic target. HNSCC dendritic-cell vaccines and innate adjuvants remain developmental, and their relevance to LSCC depends on evidence that they restore antigen-specific priming rather than inflammation alone (67, 68).

TRIM29 should also remain a Tier 3 exploratory intracellular mediator. Its inhibition could, in principle, stabilize STING or relieve PERK/ROS-associated suppression of TBK1 signaling, and SB24011 provides preclinical proof of concept for disrupting the STING-TRIM29 interaction (77–80). However, no LSCC study has established the relevant dependency, and no clinically validated TRIM29-directed intervention is available.

**Table 3** summarizes this evidence-to-intervention hierarchy and explicitly separates functional nodes, actionable mediators, and modality or classification. Combination strategies should be matched to a demonstrated bottleneck, and no LSCC-specific regimen is supported by the current evidence.

**Table 3:** Prioritized therapeutic and antibody-targeting candidates within the LSCC immune-priming framework.

Priority tier	Functional node/actionable mediator/modality or classification	Evidence basis and selection/functional readouts	Key caveat
Tier 1	Functional node: MHC-I/APM deficiency. Actionable mediator: KDM1A/LSD1. Modality/classification: candidate small-molecule epigenetic inhibition.	Direct LSCC studies show recurrent HLA-I and APM loss, providing the strongest evidence for a presentation bottleneck. Eligibility/functional readouts: tumor-cell HLA-I, B2M, TAP1/2, TAPBP, and NLRC5 status; recovered immunopeptidome and cognate T-cell recognition.	Benefit is plausible only for reversible regulatory suppression rather than irreversible genomic loss. Expression recovery must restore peptide display and cognate recognition, and no LSCC-specific therapeutic trial has validated this strategy.
Tier 1	Functional node: PD-L2-positive pDC state. Actionable mediator: PDCD1LG2/PD-L2. Modality/classification: antibody perturbation for mechanistic testing and pDC-state modulation.	Direct LSCC evidence associates PD-L2-positive pDCs with nodal disease, adverse outcome, and lower CD8 T-cell infiltration. Selection/functional readouts: cell-resolved pDC abundance, PD-L2 expression, and rescue of autologous CD8 T-cell activity after perturbation.	Causality is unproven, and PD-1 blockade already interrupts PD-L2 signaling.
Tier 1	Functional node: cGAS-STING competence. Actionable mediator: TMEM173/STING. Modality/classification: candidate agonism or targeted delivery after functional prescreening.	Direct LSCC evidence concerns tumor-cell abundance, while HNSCC and cross-tumor studies support therapeutic tractability. Selection/functional readouts: cell-resolved p-STING/p-TBK1, IFNB1 or CXCL10 output, and downstream dendritic-cell and T-cell activation.	STING abundance is not equivalent to activation, downstream output, or therapeutic response.
Tier 2	Functional node: cDC1-directed antigen delivery. Actionable mediators: CLEC9A and XCR1 as candidate receptors. Modality/classification: candidate antibody-targeted antigen-delivery design concept or cDC1-focused vaccination.	Strong cross-tumor evidence links cDC1 to cross-presentation, but LSCC cDC1 abundance and function remain unvalidated. Selection/functional readouts: lineage-resolved CLEC9A- or XCR1-positive cDC1 and defined-antigen cross-presentation.	Candidate design concept requiring dedicated LSCC-specific validation; antibody-targeting utility and functional relevance have not been validated in LSCC.
Tier 2	Functional node: cDC1 expansion and CCR7-enabled migration. Actionable mediator: FLT3L; CCR7 is retained as a migration-enabling node rather than a blockade target. Modality/classification: biologic expansion and migration-preserving dendritic-cell engineering.	Experimental evidence supports cDC1 expansion and draining-node priming. Selection/functional readouts: cDC1 expansion, CCR7-dependent migration, antigen transport to tDLN, and antigen-specific T-cell priming.	CCR7 blockade could impair priming, and LSCC-specific validation is absent.
Tier 3	Functional node: putative TRIM29-mediated suppression of STING/TBK1 output. Actionable mediator: TRIM29. Modality/classification: exploratory intracellular target for pharmacological or genetic inhibition. Mechanistic-validation priority: exploratory. Therapeutic-development priority: low/undetermined.	Viral-system studies link TRIM29 to STING degradation and to PERK/ROS-dependent inhibition of TBK1 signaling, while SB24011 provides preclinical proof of concept for disrupting the STING-TRIM29 interaction (78–80). Selection/functional readouts: cell-resolved TRIM29, STING turnover and K48-linked ubiquitination, p-TBK1/p-IRF3, IFN-I/CXCL10, and rescue after TRIM29 perturbation.	No direct LSCC evidence or clinically validated TRIM29-directed agent; TRIM29 has context-dependent cancer-promoting and tumor-suppressive functions (77–80).
Tier 3	Functional node: LAMP3/mregDC state. Actionable mediator: none established. Modality/classification: state-specific investigation or perturbation after lineage and spatial resolution.	LSCC-related single-cell studies identify LAMP3-positive states, but lineage origin and function remain uncertain. Selection/functional readouts: lineage-resolved LAMP3 state, spatial localization, migration, and cognate T-cell effects.	LAMP3 alone is not lineage specific and is not an ideal stand-alone surface target.
Tier 3	Functional node: tumor HLA-DR/CIITA axis. Actionable mediator: none established; CIITA is a candidate regulatory mediator. Modality/classification: biomarker stratification and functional perturbation or restoration studies.	Direct LSCC cohorts associate tumor HLA-DR with favorable outcome and greater CD4 T-cell infiltration. Selection/functional readouts: tumor-cell HLA-DR and CIITA status, MHC-II peptidomics, and HLA-DR-dependent cognate CD4 T-cell activation.	HLA-DR may mark an interferon-rich state and does not establish professional APC function.

### 7.2 From evidence gaps to LSCC-specific validation

Paired sampling across anatomical compartments. A multicenter prospective validation cohort would provide the clearest test of the compartment-resolved model by linking primary tumor, adjacent mucosa, blood, and anatomically mapped draining cervical nodes from the same patients. Nodal tissue is most informative when linked to metastatic status and pretreatment state. The design should preserve laryngeal subsite and record HPV testing method, tobacco and alcohol exposure, treatment history, stage, nodal basin, HLA genotype, self-reported race or ethnicity where appropriate, and genetically inferred ancestry when genomic data permit. Untreated primary disease should not be merged with treatment-exposed recurrence. Broader HNSCC evidence demonstrates site and treatment heterogeneity (1, 2), while current LSCC atlases do not connect tumor observations to priming in mapped nodes (9, 10, 66).

#### 7.2.1 Integration of molecular profiling with functional assays

Immunopeptidomics requires matched HLA typing. Fresh tissue assays should measure dendritic cell antigen uptake, maturation, costimulation, and CCR7-dependent migration. Systems using defined antigens can test cross-presentation and cognate T cell activation with peptide-loaded MHC multimers, ELISpot assays, cytokine measurements, or cytotoxicity assays. Paired tumor and node T cell receptor sequencing can identify clonotype expansion, although shared clonotypes do not prove antigen specificity. Blockade, genetic interruption, or rescue should test whether cGAS-STING, antigen-processing machinery, or a dendritic cell state is necessary for the effect. Cross-tumor studies support these measurements (19, 21, 22, 69, 70), but their LSCC relevance remains unproven.

#### 7.2.2 Validation of single-cell and spatial findings

LSCC atlases should report donor numbers and characteristics alongside cell counts, separate discovery from validation, and disclose reused datasets or overlapping specimens. Donor-level subsite, stage, treatment, handling, and batch should be reported. Cell annotations need convergent markers and orthogonal validation where possible. LAMP3-positive or mature regulatory programs should be described as states unless lineage is resolved. Ligand and receptor scores and spatial proximity generate interaction hypotheses, not evidence of signaling, antigen transfer, priming, or suppression. Trajectory and migration claims require longitudinal, lineage, or perturbational evidence (9, 10, 66).

#### 7.2.3 Candidate phenotypes remain provisional

A sensing-low phenotype would lack activation and downstream output after an appropriate stimulus. A presentation-deficient phenotype would show impaired peptide and MHC display. A regulatory-DC-dominant phenotype would inhibit cognate T cell activation in functional assays. A signal-present but functionally disconnected phenotype would show innate activation or HLA expression without migration, cross-presentation, node priming, or tumor-directed T cell output. These overlapping research categories require prespecified criteria and independent reproduction. They are not validated LSCC subtypes. **Table 4** summarizes the key validation questions, informative materials, illustrative validation approaches, decisive readouts, falsifying results, and priority levels.

**Table 4:** Key validation priorities for testing immune priming failure in laryngeal squamous cell carcinoma.

Research question	Why the gap remains	Informative material	Illustrative validation approach	Decisive readout	Result that would falsify the working hypothesis	Priority
Is the LSCC MHC-I pathway functionally defective?	Existing studies rely mainly on IHC of HLA-I and APM components	Fresh tumor, matched adjacent mucosa, blood, and when feasible tDLN	Cell-type-resolved APM profiling, immunopeptidomics, and autologous T cell recognition	Presented peptide diversity and antigen-specific activation	Normal peptide display and preserved recognition despite low IHC	High
Does tumor HLA-DR mediate presentation or mark an IFN-rich state?	HLA-DR expression and favorable outcome do not distinguish direct presentation from a correlated interferon program	Fresh HLA-DR-high and HLA-DR-low LSCC	Tumor cell purification, CIITA perturbation, MHC-II peptidomics, and autologous CD4 assays	HLA-DR-dependent antigen-specific CD4 activation	No change after HLA-DR or CIITA perturbation	High
Are functional cDC1 cells present in LSCC?	No validated LSCC cDC1 map or cross-presentation assay	Fresh tumor, invasive margin, adjacent mucosa, and tDLN	Multiparameter flow, CITE-seq, spatial profiling, and cross-presentation assay	XCR1 or CLEC9A-defined cDC1 with antigen-specific CD8 priming	cDC1 are present but do not predict or mediate cross-priming	High
Do LSCC DCs migrate to tumor-draining lymph nodes?	Tumor and lymph node compartments are rarely sampled together	Matched primary tumor and sentinel or therapeutic neck-node tissue	DC antigen transport and migration assessed using validated antigen-tracing, spatial, and state-mapping approaches, combined with parallel TCR sequencing to evaluate T-cell clonal expansion and tumor–tDLN sharing.	Shared antigen-bearing migratory DC states and newly primed T cell clones in tDLN	No tumor-derived antigen or priming signature in draining nodes	High
Do PD-L2-positive pDCs causally suppress CD8 immunity?	Current LSCC evidence is phenotypic and correlational	Fresh LSCC with high and low pDC infiltration	pDC depletion or sorting, PD-L2 perturbation, and autologous T cell assays	Restored CD8 activation after pDC or PD-L2 perturbation	No functional rescue despite efficient perturbation	Medium to high
What is the lineage origin and destination of LAMP3-positive DCs?	LAMP3 denotes a maturation state that may arise from multiple DC lineages	Normal mucosa, leukoplakia, LSCC, and matched tDLN	Joint scRNA, scATAC, CITE-seq, trajectory analysis, and spatial localization	Lineage-resolved CCR7-positive DC migration linked to priming or regulation	LAMP3 state is disconnected from migration and T cell outcomes	High
Is the cGAS-STING pathway competent in each LSCC cell compartment?	Direct LSCC data measure expression but not activation, output, or function	Fresh tumor with cell-type separation and ex vivo slices	cGAMP response, p-STING and p-TBK1, IFNB1 or CXCL10 output, and immune-cell activation	Concordant activation, interferon output, and functional immune consequences	STING-high tumors remain biochemically or functionally unresponsive	High
When does priming dysfunction emerge during laryngeal carcinogenesis?	Available precancer studies are cross-sectional	Prospective longitudinal vocal-fold leukoplakia cohort with serial sampling	Spatial immune profiling, clonotype tracking, and progression-linked pathology	Within-patient transition in presentation, DC state, and antigen-specific T cell clonotypes	No reproducible temporal change before invasion	High
Can a compartment-resolved biomarker predict response to immunotherapy?	Single markers such as HLA-DR or STING are not validated predictive biomarkers in LSCC	Prospective LSCC immunotherapy cohort with pretreatment and on-treatment tissue	Composite tumor APM, DC state, tDLN, interferon output, and TCR metrics	Predefined association with response and external validation	No incremental value beyond standard clinical biomarkers	Medium to high
Which innate adjuvant or DC intervention restores priming?	Preclinical activation can fail when tumor or myeloid STING is dysfunctional	Patient-derived organotypic cultures plus immunocompetent LSCC models	Functional prescreening followed by STING agonist, Flt3L, DC vaccine, or radiation combinations	Antigen-specific priming and durable tumor control with toxicity monitoring	Inflammation without antigen-specific priming or tumor control	Medium

### 7.3 Computational, in silico, and AI-enabled strategies

The compartment-resolved model creates a multimodal data-integration problem that is well suited to bioinformatics, provided that computational inference remains anchored to explicit biological questions. HLA-aware neoantigen and immunopeptidome prediction can prioritize peptides for experimental testing. Paired T cell receptor repertoire analysis can identify clonotypes shared between tumor-draining lymph nodes and tumors, whereas single-cell and spatial pipelines can resolve dendritic-cell states, tissue neighborhoods, and candidate interactions. Recent HNSCC literature highlights multi-omics integration as a route to identify immunotherapy biomarkers and therapeutic targets (73), and spatial transcriptomics combined with artificial intelligence is emerging in head and neck pathology (74). In silico peptide-HLA binding, neoantigen prioritization, and T cell receptor specificity prediction can narrow the experimental search space, but they cannot replace immunopeptidomics or antigen-specific functional assays.

Artificial intelligence and machine learning are most informative when they integrate pathology, spatial transcriptomics, HLA genotype, immunopeptidomics, T cell receptor features, and clinical variables rather than converting a single omics layer into an opaque score. Cross-tumor work has shown that HLA-informed spatial and multi-omics features can be integrated into predictive models, providing proof of concept but not LSCC validation (75). Broader cancer immunotherapy literature similarly emphasizes multimodal integration, interpretability, prospective testing, and generalizability as prerequisites for clinical translation (76). For LSCC, discovery models should use patient-level cross-validation, preserve center and population structure, report calibration and uncertainty, and undergo external validation in independent cohorts. Biological features that drive prediction should also be tested experimentally. AI-derived signatures should therefore remain hypothesis-generating until they reproduce across centers and improve prediction beyond standard clinical and immune biomarkers.

### 7.4 Evidence limitations and interpretive boundaries

The principal limitation is the small and fragmented LSCC-specific evidence base. Several direct studies are single-center retrospective series that use immunohistochemistry, flow cytometry, or ex vivo monocyte-derived dendritic cells to measure isolated pathway nodes (3–6, 8, 11, 39–41). These designs can establish abundance, spatial association, or prognosis within their sampled populations. They cannot establish antigen specificity, the anatomical site of priming, or a continuous causal sequence.

A second limitation is transfer across anatomical sites and experimental systems. Mixed-site HNSCC studies inform biological plausibility, but site composition, HPV status, treatment exposure, and sampling compartment may differ from LSCC (1, 2, 7, 12). Mechanistic studies in other tumors identify possible roles for cDC1, CCR7, tumor-draining lymph nodes, and cGAS-STING signaling, but they do not establish that the same bottleneck exists in laryngeal cancer. This Review therefore uses cross-site evidence to define tests, not to fill missing LSCC results.

Third, current single-cell and spatial studies remain vulnerable to small donor numbers, cross-sectional pseudotemporal inference, reuse of public datasets, and incomplete functional validation (9, 10, 66). Cell counts cannot substitute for independent biological donors. Transcriptomic state labels, ligand and receptor scores, and spatial proximity do not demonstrate lineage, migration, antigen transfer, or suppression. No study in the current evidence set pairs a primary LSCC with anatomically mapped draining nodes and directly tracks tumor antigen, cognate T cell activation, and clonotype movement across compartments.

A further limitation is the absence of adequately powered analyses of host immunogenetics and population diversity. HLA allele distributions differ among populations, and HLA genotype can alter the repertoire available for antigen presentation, but no current LSCC dataset establishes how these variables modify candidate priming phenotypes (71, 72). Self-reported race or ethnicity, genetic ancestry, HLA genotype, environmental exposure, and healthcare context should therefore be analyzed separately rather than treated as interchangeable proxies. This gap also limits the transportability of computational models trained in geographically restricted cohorts and reinforces the need for multicenter validation across diverse populations.

Finally, the translational categories proposed here have not been prospectively validated. Tumor HLA-DR has prognostic and immune-context associations, but no interaction between treatment and biomarker status has been demonstrated in LSCC (5, 6). PD-L2-positive pDCs and tumor cell STING abundance remain correlates rather than causal or predictive markers (8, 11). The proposed sensing-low, presentation-deficient, regulatory-DC-dominant, and signal-present but functionally disconnected phenotypes are research constructs. They are not diagnostic classes, enrollment biomarkers, or treatment recommendations.

## 8 Conclusions

LSCC contains several observations that make defective immune priming a credible but unproven research question. These include loss of MHC class I antigen-processing components, favorable associations with tumor HLA-DR, accumulation of PD-L2-positive pDCs, altered dendritic-cell states, and stage-associated tumor-cell STING abundance (3–6, 8–11). Yet no LSCC study identified in this Review demonstrates the complete sequence from innate danger sensing and peptide presentation to dendritic-cell migration, tumor-draining lymph-node priming, and antigen-specific T cell entry into the tumor. The compartment-resolved model should therefore be read as an evidence-organizing and hypothesis-testing framework, not as an established LSCC-specific mechanism.

Progress will require compartment-resolved and temporally defined studies that pair tumor tissue with adjacent mucosa, blood, and anatomically mapped draining lymph nodes. Molecular atlases should be linked to immunopeptidomics, dendritic-cell function, perturbation or rescue experiments, and antigen-specific T cell assays. Among translational candidates, the MHC-I antigen-processing machinery has the strongest direct LSCC evidence for a potentially reversible presentation bottleneck. PD-L2-positive pDC states and cell-resolved STING competence merit high-priority mechanistic validation, whereas CLEC9A, XCR1, FLT3L, and related cDC1 strategies are compelling but currently supported mainly by HNSCC or cross-tumor evidence. Multicenter studies should incorporate HLA genotype and population diversity, and computational or AI models should be externally validated and linked to functional assays. Until these findings are prospectively reproduced, the proposed pathway nodes and priming phenotypes should remain research variables rather than clinical selection tools. TRIM29 is an exploratory intracellular modifier of STING/TBK1 output, but its expression, pathway dependence, and therapeutic tractability remain untested in LSCC.
